# *In Vitro* Antitumour Activity of Some Triorganophosphinegold(I) Thionucleobases

**DOI:** 10.1155/MBD.1994.299

**Published:** 1994

**Authors:** Edward R. T. Tiekink, Peter D. Cookson, Bernadette M. Linahan, Lorraine K. Webster

**Affiliations:** 1 Department of Chemistry The University of Adelaide Adelaide South Australia 5005 Australia; 2 Experimental Chemotherapy and Pharmacology Unit Peter MacCallum Cancer Institute 481 Little Lonsdale Street Melbourne Victoria 3000 Australia

## Abstract

A series of phosphinegold(I) thionucleobase analogues, [R_3_PAu(SR^x^)] (R = Et, Ph or chexyl;  HSR^1^ = 2-mercaptobenzoic acid, HSR^2^ = 2-thiouracil, HSR^3^ = 6-mercaptopurine and HSR^4^ = 6-thioguanine) have been examined for their *in vitro* cytotoxicity in L1210 murine leukemia cells in culture. The range of ID_50_ values (continuous 48 h exposure) for the complexes is 0.041 - 0.131 μM. The complexes with SR^3^ and SR^3^ are generally the most active; however, there is no clear trend associated with the phosphine ligands.


### *IN VITRO* ANTITUMOUR ACTIVITY OF SOME TRIORGANOPHOSPHINEGOLD(I) THIONUCLEOBASES

Edward R.T. Tiekink*[1], Peter D. Cookson[1],
Bernadette M. Linahan[2] and Lorraine K. Webster*[2]

[1] Department of Chemistry, The University of Adelaide, Adelaide, South Australia 5005, Australia
[2] Experimental Chemotherapy and Pharmacology Unit,
Peter MacCallum Cancer Institute, 481 Little Lonsdale Street, Melbourne, Victoria 3000, Australia

**Abstract.**

A series of phosphinegold(I) thionucleobase analogues, $[R_3PAu(SR^x)]$ (R = Et, Ph or c-hexyl; $HSR^1$ = 2-mercaptobenzoic acid, $HSR^2$ = 2-thiouracil, $HSR^3$ = 6-mercaptopurine and $HSR^4$ = 6-thioguanine) have been examined for their *in vitro* cytotoxicity in L1210 murine leukemia cells in culture. The range of $ID_{50}$ values (continuous 48 h exposure) for the complexes is 0.041 - 0.131 μM. The complexes with $SR^3$ and $SR^4$ are generally the most active; however, there is no clear trend associated with the phosphine ligands.

**Introduction.**

Transition metal complexes have proved to possess significant anticancer activity and there are several reviews summarizing recent progress in this field.[1] Most notable among these complexes are those containing platinum, although other metal species, e.g. Ru(II), Rh(II), Rh(III) and Au(I), also show promising activity. Gold complexes have a long history in medicine and several complexes are used in the treatment of rheumatoid arthritis at present.[2] Given the clinical success of Au(I) complexes it is not surprising that the antitumor activity of some of these complexes has been investigated.[3-5] The monomeric species Auranofin, (1-thio-β-D-gluco-pyranose 2,3,4,6-tetraacetato-*S*)(triethyl-phosphine)gold(I), an anti-arthritic drug, has been shown to possess maximum activity against P388 leukemia;[3] however, it was the bis-chelated diphosphine Au(I) complex, $[Au(Ph_2P(CH_2)_2PPh_2)_2]^+$, that showed the most promising activity.[5] Certain phosphinegold(I) thiolates, i.e. containing the P-Au-S arrangement as in Auranofin, also show encouraging activity[4,6] and the most notable among these is $Ph_3PAu$(8-

thiotheophyllinate).[6] As an extension of previous studies of the anti-arthritic activity of related complexes,[7] the *in vitro* antitumor activity of a series of phosphinegold(I) thiolates, where the thiolate ligand is derived from a thionucleobase, has been investigated and the results of this study are reported herein. The antitumor activity of two of the complexes has been reported previously in a conference abstract; however, to our knowledge, no subsequent results have been published.[8]

### Results and Discussion

*Chemistry.* The $[R_3PAu(SR')]$ complexes have been prepared by the reaction of $[R_3PAuCl]$ and the thionucleobase in the presence of KOH. The complexes have been characterized spectroscopically and in the case of certain examples, using X-ray crystallographic techniques.[7,9] The colorless/off-white solids are obtained in good yields and are relatively air- and light-stable; only the $[Et_3PAu(SR^1)]$ complex proved too unstable for testing. The thiolates chosen for this study, shown below, are the deprotonated forms of 2-mercaptobenzoic acid ($HSR^1$), 2-thiouracil ($HSR^2$), 6-mercaptopurine ($HSR^3$) and 6-thioguanine ($HSR^4$); the R groups bound to the phosphine ligands are Et, Ph or c-hexyl.

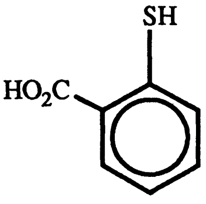

$HSR^1$

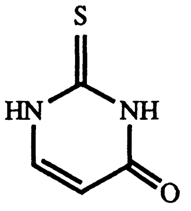

$HSR^2$

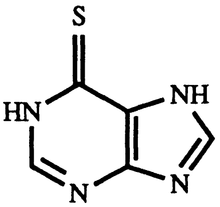

$HSR^3$

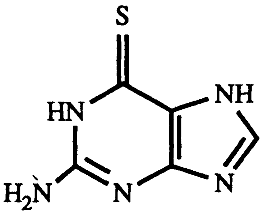

$HSR^4$

Crystal structure determinations on the complexes have revealed the presence of a linear P-Au-S arrangement and a thiolate ligand (rather than a thione) in each case; an illustration of the molecular structure of a representative compound, i.e. $[Ph_3PAu(SR^3)]$, as determined by X-ray diffraction methods is shown in Figure 1.[7b]

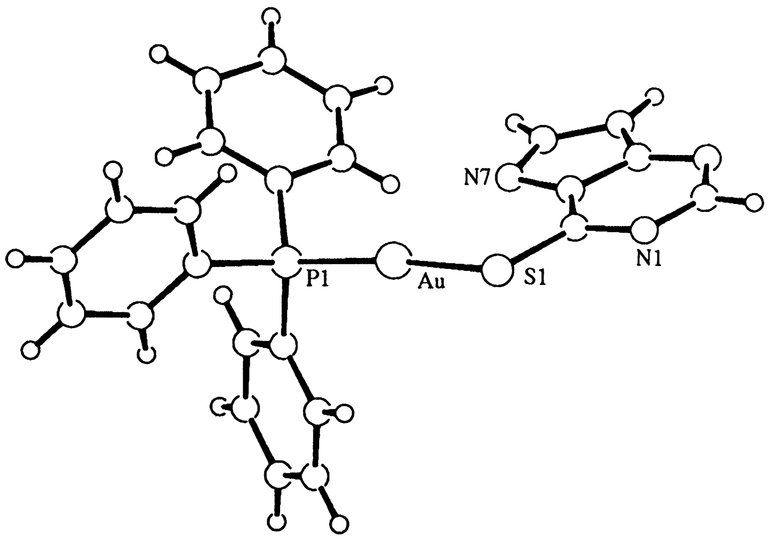

Figure 1 The molecular structure of $[Ph_3PAu(SR^3)]$.[7b]

*Biological Testing.* The results of growth inhibition testing of the phosphinegold(I) thionucleobase complexes against murine L1210 leukemia cells in culture are summarized in Table 1. It is noteworthy that the presence of gold in the complexes generally results in higher activity than that exhibited by the free thionucleobases, two of which possess significant antitumor activity: 6-mercaptopurine ($HSR^3$) and 6-thioguanine ($HSR^4$) are used clinically for the treatment of leukemia.[10] The complexes of $HSR^3$ resulted in at least three-fold greater activity, although the addition of gold to 6-thioguanine did not increase significantly its growth inhibitory properties. For the 2-mercaptobenzoic acid ($HSR^1$) and 2-thiouracil ($HSR^2$) derivatives, the activity was enhanced by at least two to three orders of magnitude, pointing to the critical role of Au(I). The only metal-containing anticancer drugs in common use are cisplatin and carboplatin; for comparison, their $ID_{50}$'s against L1210 are approximately 0.6 and 12 μM, respectively.

An examination of the $ID_{50}$ values in Table I reveals that no simple structure/activity relationship exists between the nature of the phosphine ligand and the *in vitro* activity against the L1210 murine leukemia. In fact, there is no significant difference in activity among the complexes within any one group. Nevertheless, all of the compounds are at least as active as 6-thioguanine, and represent a potentially exciting new class of antitumor agent. Further work is underway to determine the range of activity in other tumor types, and to investigate the mechanism of action.

**Table 1.** Biological Activity of the Thiols ($HSR^x$) and the Phosphinegold(I) Thiolates ($[R_3PAu(SR^x)]$) [a]

| Thiols | $HSR^1$ | $HSR^2$ | $HSR^3$ | $HSR^4$ |
|---|---|---|---|---|
| | > 20 | > 100 | 0.310±0.133 | 0.096±0.022 |
| Complexes | $SR^1$ | $SR^2$ | $SR^3$ | $SR^4$ |
| R = Et | - | 0.086±0.082 | 0.094±0.051 | 0.041±0.013 |
| R = Ph | 0.047±0.005 | 0.131±0.072 | 0.083±0.048 | 0.052±0.017 |
| R = c-hexyl | 0.082±0.022 | 0.115±0.020 | 0.079±0.028 | 0.084±0.030 |

a Values quoted are $ID_{50}$ (μM) and represent the dose of compound causing 50% growth inhibition of L1210 mouse leukemia cells in culture after 48 h exposure to the compound. Results are expressed as mean ± standard deviation of three to five replicates.

### Experimental Section

*(a) Syntheses.* The complexes and their characterization are as reported in earlier papers.[7,9]

*(b) Biological Testing.* L1210 mouse leukemia cells were grown as suspension cultures in Eagle's minimum essential medium plus 1% glutamine and 10% fetal calf serum (Flow Laboratories). The compounds were dissolved in DMSO at 10 concentrations over a 3-log range, with a final maximum DMSO concentration in the medium of 0.5%. Growth inhibition was tested by incubation of log-phase cells at 37°C in a humidified incubator gassed with 10% $CO_2$/90% air for 48 h in the presence of the compound. Cells were then counted using a Coulter counter, and the $ID_{50}$ in μM, or dose causing 50% inhibition of cell growth, was determined from the curve of percentage growth versus dose. All assays were performed at least in triplicate. Control cultures exposed only to the vehicle were the reference for 100% growth in each test.

**Acknowledgments.** The support of this research by the Australian Research Council is gratefully acknowledged.

### References

( 1 ) (a) Köpf-Maier, P.; Köpf, H. Non-platinum-group metal antitumor agents: History, current status and perspectives. *Chem. Rev.*, **1987**, *87*, 1137; (b) Keppler, B.K. Metal complexes as anticancer agents. The future role of inorganic chemistry in cancer therapy. *New J. Chem.*, **1990**, *14*, 389; (c) Haiduc, I.; Silvestru, C. Metal compounds in cancer chemotherapy. *Coord. Chem. Rev.*, **1990**, *99*, 253.

( 2 ) (a) Lewis, A.J.; Walz, D.T. Immunopharmacology of gold. *Prog. Med. Chem.*, **1982**, *19*, 1; (b) Sutton, B.M. Gold compounds for rheumatoid arthritis. *Gold* Bull., **1986**, *19*, 15; (c) Parish, R.V. Gold in medicine - chrysotherapy. *Interdisciplinary Sci. Rev.*, **1992**, *17*, 221; Tiekink, E.R.T.; Whitehouse, M.W. The use of gold compounds in medicine. *Chem. Aust.*, **1990**, *57*, 346.

( 3 ) (a) Simon, T.M.; Kunishima, D.H., Vibert, G.J.; Lorber, A. Screening trial with the coordinated gold compound auranofin using mouse lymphocytic leukemia P388. *Can. Res.*, **1981**, *41*, 94; (b) Mirabelli, C.K.; Johnson, R.K.; Sung C.-M.; Faucette, L.F.; Muirhead, K.; Crooke, S.T. Evaluation of the in vivo antitumor activity and in vitro cytotoxic properties of auranofin, a coordinated gold compound, in murine tumor models. *Cancer Res.*, **1985**, *45*, 32.

( 4 ) Mirabelli, C.K.; Johnson, R.K.; Hill, D.T.; Faucette, L.F.; Girard, G.R.; Kuo, G.Y.; Sung C.-M.; Crooke, S.T. Correlation of the in vitro cytotoxic and in vivo antitumor activities of gold(I) coordination complexes. *J. Med. Chem.*, **1986**, *29*, 218.

( 5 ) (a) Berners-Price, S.J.; Mirabelli, C.K.; Johnson, R.K.; Mattern, M.R.; McCabe, F.L.; Faucette, L.F.; Sung, C.-M.; Mong, S.-M.; Sadler, P.J.; Crooke, S.T. In vivo antitumor activity and in vitro cytotoxic properties of bis[1,2-bis(diphenylphosphino)ethane]gold(I) chloride. *Can. Res.*, **1986**, *46*, 5486; (b) Mirabelli, C.K.; Hill, D.T.; Faucette, L.F.; McCabe, F.L.; Girard, G.R.; Bryan, D.B.; Sutton, B.M.; Bartus, J.O.; Crooke, S.T.; Johnson, R.K. Antitumor activity of bis(diphenylphosphino)alkanes, their gold(I) coordination complexes, and related compounds. *J. Med. Chem.*, **1987**, *30*, 2181; (c) Berners-Price, S.J.; Girard, G.R.; Hill, D.T.; Sutton, B.M.; Jarrett, P.S.; Faucette, L.F.; Johnson, R.K.; Mirabelli, C.K.; Sadler, P.J. Cytotoxicity and antitumor activity of some tetrahedral bis(diphosphino)gold(I) chelates. *J. Med. Chem.*, **1990**, *33*, 1386.

( 6 ) Arizti, M.P.; Garcia-Orad, A.; Sommer, F.; Silvestro, L.; Massiot, P.; Chevallier, P.; Gutiérrez-Zorrilla; Colacio, E.; Martinez de Pancorbo, M.; Tapiero, H. Intracellular accumulation and cytotoxic effect of (8-thiotheophyllinate)(triphenylphosphine)gold(I) in Friend leukemia cells. *Anticancer Res.*, **1991**, *11*, 625.

( 7 ) (a) Harker, C.S.W.; Tiekink, E.R.T.; Whitehouse, M.W. Studies on the interaction of gold(I) phosphines with 2-thiouracil. Related studies with silver(I) phosphines. *Inorg. Chim. Acta*, **1991**, *181*, 23; (b) Cookson, P.D.; Tiekink, E.R.T.; Whitehouse, M.W. Phosphinegold(I) complexes containing the 6-mercaptopurinate anion, and their anti-arthritic activity. *Aust. J. Chem.*, **1994**, *47* in press.

( 8 ) Agrawal, K.C.; Bears, K.B.; Marcus, D.; Jonassen, H.B.; Gold triphenylphosphine complexes as a new class of potential antitumor agents. *Proc. Am. Assoc. Can. Res.*, **1978**, *18*, 28.

( 9 ) Cookson, P.D.; Tiekink, E.R.T. Syntheses and structural studies of triorganophosphinegold(I) mercaptobenzoate complexes. *J. Coord. Chem.*, **1993**, *26*, 313.

( 1 0 ) McCormack, J.J.; Johns, D.G. Purine and purine nucleoside antimetabolites. *in* Cancer Chemotherapy: Principles and Practice, Chabner, B.A., Collins, J.M. (Eds) Lippincott Company, Philadelphia, 1990, Chapter 9.

**Received: January 17, 1994 - Accepted: February 24, 1994**

